# Draft Genome Assembly of *Delftia acidovorans* Type Strain 2167

**DOI:** 10.1128/genomeA.00917-14

**Published:** 2014-09-18

**Authors:** K. W. Davenport, H. E. Daligault, T. D. Minogue, K. A. Bishop-Lilly, D. C. Bruce, P. S. Chain, S. R. Coyne, K. G. Frey, J. Jaissle, G. I. Koroleva, J. T. Ladner, G. F. Palacios, C. L. Redden, M. B. Scholz, H. Teshima, S. L. Johnson

**Affiliations:** a Los Alamos National Laboratory, Los Alamos, New Mexico, USA; b USAMRIID-DSD, Fort Detrick, Maryland, USA; c NMRC-Frederick, Fort Detrick, Maryland, USA; d Henry M. Jackson Foundation, Bethesda, Maryland, USA; e Center for Genome Sciences (CGS), United States Army Medical Research Institute of Infectious Diseases, Fort Detrick, Maryland, USA

## Abstract

The *Delftia acidovorans* 2167 (ATCC 15668, *Delftia* type strain) genome was sequenced into a 6-contig scaffolded assembly of 6.78-Mb. This environmental microbe, previously named to both the *Comamonas* and *Pseudomonas* genera, is an opportunistic pathogen and often the subject of phylogenetic placement debates.

## GENOME ANNOUNCEMENT

A common microbe of soils and freshwater and originally isolated in Delft, Netherlands, *Delftia acidovorans* is an opportunistic pathogen of immunocompromised individuals (1–4). Infections are rare, but rapid identification is imperative as many are resistant to aminoglycosides (5). We sequenced the genome of *Delftia acidovorans* 2167 (ATCC 15668, *Delftia* type strain), isolated in 1926 from acetamide-enriched soil.

High-quality genomic DNA was extracted from a purified isolate using a Qiagen Genomic-tip 500 at USARMIID-DSD. Specifically, a 100-mL bacterial culture was grown to stationary phase and nucleic acid extracted per the manufacturer’s recommendations. Sequence data for the draft genome include a combination of Illumina and 454 technologies (6, 7). We constructed and sequenced an Illumina library of 100-bp reads to 448-fold genome coverage and a separate long-insert paired-end library (21-fold genome coverage, 6,961 ± 1,741-bp insert) (Roche 454 Titanium platform). The two datasets were assembled together in Newbler (Roche) and the consensus sequences were computationally shredded into 2-kbp overlapping fake reads (shreds). Raw reads were also assembled in Velvet and those consensus sequences were computationally shredded into 1.5-kbp overlapping shreds (8). All draft data were then assembled together with Allpaths and the consensus sequences computationally shredded into 10-kbp overlapping shreds (9). Finally, we used parallel Phrap (High Performance Software, LLC) to integrate the Newbler consensus shreds, Velvet consensus shreds, Allpaths consensus shreds, and a subset of the long-insert read-pairs . Possible misassemblies were corrected and some gap closure accomplished with manual editing in Consed (10–12).

Automatic annotation for the *D. acidovorans* 2167 genome utilized an Ergatis-based workflow at LANL with minor manual curation. The 6,777,458-bp annotated genome contains 66.6% G+C and 6,043 coding sequences. The final scaffolded assembly (6 contigs) is available in NCBI and raw data files are available upon request.

### Nucleotide sequence accession number.

This genome is available in GenBank under accession number JOUB00000000.
